# Impact of the menstrual cycle on commercial prognostic gene signatures in oestrogen receptor-positive primary breast cancer

**DOI:** 10.1007/s10549-021-06377-3

**Published:** 2021-09-15

**Authors:** Ben P. Haynes, Gene Schuster, Richard Buus, Anastasia Alataki, Ophira Ginsburg, Le Hong Quang, Pham Thi Han, Pham Hong Khoa, Nguyen Van Dinh, Ta Van To, Mark Clemons, Chris Holcombe, Caroline Osborne, Abigail Evans, Anthony Skene, Mark Sibbering, Clare Rogers, Siobhan Laws, Lubna Noor, Maggie Chon U. Cheang, Susan J. Cleator, Ian E. Smith, Mitch Dowsett

**Affiliations:** 1 Ralph Lauren Centre for Breast Cancer Research, Royal Marsden Hospital, Fulham Road, London, SW3 6JJ UK; 2 The Breast Cancer Now Toby Robins Research Centre, The Institute of Cancer Research, Fulham Road, London, SW3 6JB UK; 3 Department of Medicine, University of Toronto, Toronto, Canada; 4 Department of Population Health, Perlmutter Cancer Center, NYU Langone Health, New York, USA; 5 Department of Breast Surgery, National Cancer Hospital, Hanoi, Vietnam; 6 Department of Pathology, National Cancer Hospital, Hanoi, Vietnam; 7 Division of Medical Oncology, Department of Medicine, The Ottawa Hospital and University of Ottawa, Ottawa, Canada; 8 Royal Liverpool University Hospital, Prescott Street, Liverpool, UK; 9 Yeovil District Hospital, Yeovil, Somerset UK; 10 Poole Hospital, Longfleet Road, Poole, Dorset UK; 11 Royal Bournemouth Hospital, Castle Lane East, Bournemouth, Dorset UK; 12 Royal Derby Hospital, Uttoxeter Road, Derby, UK; 13 Doncaster Royal Infirmary, Armthorpe Road, Doncaster, South Yorkshire UK; 14 Royal Hampshire County Hospital, Winchester, Hampshire UK; 15 University Hospital North Tees, Hardwick Road, Stockton-on-Tees, UK; 16 Clinical Trials and Statistics Unit, Institute of Cancer Research, London, UK; 17 Department of Clinical Oncology, Imperial Healthcare NHS Trust, London, UK; 18 The Breast Unit, Department of Medicine, Royal Marsden Hospital, Fulham Road, London, UK

**Keywords:** Breast cancer, Oestrogen-regulated genes, Menstrual cycle, Prognostic signatures, Hormone receptors

## Abstract

**Purpose:**

Changes occur in the expression of oestrogen-regulated and proliferation-associated genes in oestrogen receptor (ER)-positive breast tumours during the menstrual cycle. We investigated if Oncotype® DX recurrence score (RS), Prosigna® (ROR) and EndoPredict® (EP/EPclin) prognostic tests, which include some of these genes, vary according to the time in the menstrual cycle when they are measured.

**Methods:**

Pairs of test scores were derived from 30 ER-positive/human epidermal growth factor receptor-2-negative tumours sampled at two different points of the menstrual cycle. Menstrual cycle windows were prospectively defined as either W1 (days 1–6 and 27–35; low oestrogen and low progesterone) or W2 (days 7–26; high oestrogen and high or low progesterone).

**Results:**

The invasion module score of RS was lower (− 10.9%; *p* = 0.098), whereas the ER (+ 16.6%; *p* = 0.046) and proliferation (+ 7.3%; *p* = 0.13) module scores were higher in W2. *PGR* expression was significantly increased in W2 (+ 81.4%; *p* = 0.0029). Despite this, mean scores were not significantly different between W1 and W2 for any of the tests and the two measurements showed high correlation (*r* = 0.72–0.93). However, variability between the two measurements led to tumours being assigned to different risk categories in the following proportion of cases: RS 22.7%, ROR 27.3%, EP 13.6% and EPclin 13.6%.

**Conclusion:**

There are significant changes during the menstrual cycle in the expression of some of the genes and gene module scores comprising the RS, ROR and EP/EPclin scores. These did not affect any of the prognostic scores in a systematic fashion, but there was substantial variability in paired measurements.

**Supplementary Information:**

The online version contains supplementary material available at 10.1007/s10549-021-06377-3.

## Introduction

Oestrogen receptor (ER)-positive disease represents approximately 80% of breast cancers [1, 2]. Standard treatment of patients with ER-positive disease comprises surgery and adjuvant endocrine therapy with the addition of chemotherapy based on clinical risk factors and/or prognostic estimates from one of several gene expression-based tools. Three of the most widely used tumour profiling tests are the Oncotype DX Recurrence Score (RS) [3], Prosigna risk of recurrence (ROR) score often known as the PAM50 [4] and EndoPredict (EP/EPclin) [5], which provide an estimate of the 10-year risk of distant recurrence assuming 5 years of adjuvant endocrine therapy without chemotherapy and are endorsed for use in ER-positive, human epidermal growth factor receptor-2 (HER2)-negative and lymph node-negative disease in authoritative guidelines [6, 7]. The data supporting their use are stronger in postmenopausal than premenopausal patients although they are applied clinically in both settings.

RS comprises 16 prognostic genes and five reference genes measured by RT-PCR at a central laboratory (Genomic Health, CA, USA). The RS algorithm creates four modules (proliferation, oestrogen, HER2 and invasion) from 13 of the prognostic genes [8]. This generates a RS result of between 0 and 100, which relates to the 10-year risk of distant recurrence in the absence of chemotherapy. Cut-points of < 18, 18–31 and > 31 are applied to classify patients into low-, intermediate- and high-risk groups [3]. The TAILORx study provided evidence for lower cut-points (intermediate group 11–25), which are now widely applied [9]. The RS can be combined with clinical and pathological factors generating tools, such as the RS-pathology-clinical (RSPC) [10] and the RSClin [11].

The ROR is a 50 gene (plus eight reference genes) test performed on the NanoString nCounter platform [4, 12]. In addition to a continuous risk score (0–100), the test provides intrinsic subtype classification (Luminal A or B, HER-enriched, Basal-like). The ROR is calculated from the correlation of the expression profile of the sample with the reference gene expression profile (centroid) for each intrinsic subtype, combined with a score from the proliferative genes and tumour size [4, 12]. Risk categories are defined by cut-points of 0–40 (low), 41–60 (intermediate) and 61–100 (high) for node-negative cancers and 0–15 (low), 16–40 (intermediate) and 41–100 (high) for one to three node-positive cancers.

The EP score represents the molecular component of EPclin and comprises eight prognostic genes and four reference genes [5]. The test is RT-PCR based. The EP score ranges between 0 and 15 and uses a cut-point of 5 to categorise patients into low- and high-risk groups. EPclin, the read-out of the clinically available EndoPredict test, combines the EP score with tumour size and nodal status and ranges between 0 and 8.16 with a cut-point of 3.3 used to categorise patients into low- and high-risk groups [5].

Each of the above tests includes a number of oestrogen-responsive genes (ERGs) and proliferation-associated genes (PAGs). The expression of some ERGs and PAGs in ER-positive breast cancers is known to vary across the menstrual cycle [13, 14]. A recent study found significant changes in the expression of ERGs (twofold to threefold) and PAGs (1.4-fold) within the same patient that related to the hormone changes that occur during the menstrual cycle [15].

The presence of multiple ERGs and PAGs within the commercial signatures suggests that these tests may be sensitive to the prevailing hormone milieu at the time of testing. Theoretically, this might lead to a different score and risk categorisation being obtained depending on the point of the menstrual cycle when the prognostic signature was measured. Thus, we have investigated if RS, ROR and EP/EPclin scores vary according to the time in the menstrual cycle when they are measured.

## Materials and methods

### Patients and samples

Samples were selected from two clinical trials reported in a recent study of the effect of the menstrual cycle on breast tumour biology in ER-positive breast cancer [15]: MenCER, a UK-based multicentre study [15] and a study of neoadjuvant oophorectomy in Vietnam [16]. Paired tumour samples were taken at diagnosis and 1–4 weeks later, with no treatment occurring between these time-points.

In the current study, samples were assigned to two menstrual cycle windows, based on their previously measured serum hormone concentrations and menstrual cycle data [15]: Window 1 (W1; early and very late cycle; days 1–6 and 27–35) when there are low levels of both oestradiol and progesterone and Window 2 (W2; mid and late cycle; days 7–26) when there are intermediate to high levels of oestradiol and low to high levels of progesterone. Based on these criteria, 22 patients were available with paired W1 and W2 tumour samples from which RNA was taken. Eight further patients where RNA was available from paired tumour samples taken in the same window (2 × W1 vs. W1, 6 × W2 vs. W2) were selected as control samples.

Ethical approval for MenCER was received from the local research ethics committee (South West London REC 3). The Vietnamese study was approved by the Institutional Ethics Committee of the National Cancer Hospital, Hanoi, Vietnam from where all study participants were recruited and by the Research Ethics Board of the University of Toronto, Canada from where the study was coordinated. All participants provided written informed consent. The Committee for Clinical Research at the Royal Marsden Hospital, London approved the analysis of the samples collected in this study.

### Measurement of gene expression

The NanoString nCounter gene expression system (GEN2) (NanoString Technologies, Seattle, WA) was used to measure gene expression without target amplification [17]. A custom gene expression nCounter CodeSet was used to measure the expression of 82 genes including 14 reference genes (Supplementary Table 1) that include the genes of the RS, ROR and EP prognostic signatures. In brief, the CodeSet was hybridised to 150–200 ng total RNA and samples were processed using the NanoString nCounter Prep Station and Digital Analyzer according to the manufacturer’s instructions.

### Calculation of RS, ROR and EP/EPclin scores and % risk of distant recurrence

The gene expression normalisation and adjustment factors of NanoString data used to calculate the ‘research use only’ (RUO) RS and EP scores are described in Buus et al. [18]. Briefly, validated linear models were used to adjust each signature gene for cross-platform (NanoString vs. RT-PCR) variation and to generate RUO scores according to their published algorithms [3, 5]. RUO EPclin scores were calculated from RUO EP scores using the EPclin algorithm [5] incorporating tumour size and nodal status. The corresponding % risk of distant recurrence at 10 years was calculated for RS using web-based tools provided by GHI [19] and for EP/EPclin by digital read-out (https://apps.automeris.io/wpd/) from the published graphs of EP/EPclin score vs. % risk [5]. RUO ROR scores and their corresponding % risk of distant recurrence at 10 years were calculated by NanoString.

### Data analysis

For paired data, the Wilcoxon matched-pairs signed rank test was used to compare differences in gene expression. For individual genes, false discovery rate was calculated using the Benjamini–Hochberg procedure to adjust for multiple testing. The F test was used to compare variances of the different scores and risks in paired samples taken in either different or the same window. To study associations between continuous variables Spearman’s rank correlation was used.

## Results

### Patient demographics

Patient demographics of the 30 patients are described in Supplementary Table 2. All patients were premenopausal with ER-positive/HER2-negative tumours. Of those, 88% were progesterone receptor (PgR)-positive and 8 patients had node-positive disease (range 1–2 nodes positive).

### Changes in RS, ROR and EP scores during the menstrual cycle

**Fig. 1:**
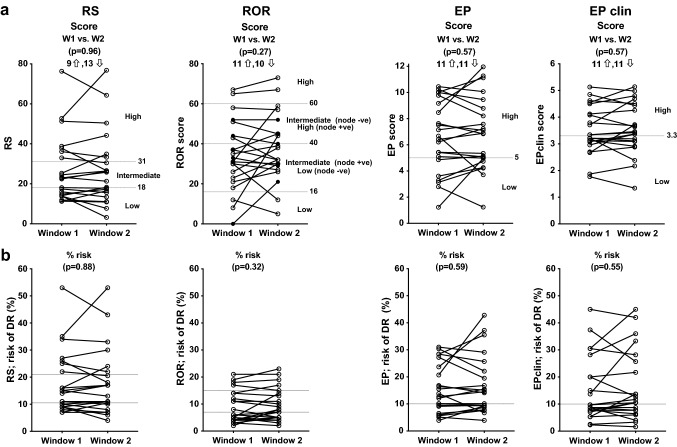
Changes in **a** RS, ROR, EP & EPclin scores and **b** % risk estimates of distant relapse, in paired tumour samples taken in W1 (low oestrogen and progesterone) vs. W2 (high oestrogen ± progesterone). Dotted lines indicate cut-points between risk categories

Figure 1a shows the individual changes in the prognostic scores between W1 (low oestrogen and progesterone) and W2 (high oestrogen ± progesterone) for each test. Mean [± standard error of the mean (SEM)] scores were not significantly different between W1 and W2 for RS (26.7 ± 3.5 vs. 26.9 ± 3.9; Wilcoxon *p* = 0.96), ROR (34.2 ± 3.7 vs. 38.0 ± 3.6; *p* = 0.27), EP (6.57 ± 0.58 vs. 6.82 ± 0.59; *p* = 0.57) or EPclin (3.50 ± 0.19 vs. 3.57 ± 0.20; *p* = 0.57) (Fig. 1a). There was a strong correlation of the individual signature scores in W1 and W2 with ROR showing the largest variation (RS; *r* = 0.93, ROR; *r* = 0.72, EP; *r* = 0.85, EPclin; *r* = 0.82; Supplementary Fig. 1a). The mean (± SEM) absolute difference in scores between W1 and W2 irrespective of direction of change was 5.2 ± 1.1 for RS, 9.2 ± 2.0 for ROR, 1.18 ± 0.25 for EP and 0.33 ± 0.07 for EPclin.

The change in the corresponding estimates of % risk of recurrence generated from each score is shown in Fig. 1b; again, there was no significant difference between W1 and W2 for RS (mean ± SEM, 17.7 ± 2.5% vs. 17.9 ± 2.7%; *p* = 0.88), ROR (8.9 ± 1.3% vs. 9.8 ± 1.3%; *p* = 0.32), EP (15.6 ± 1.9% vs. 16.8 ± 2.3%; *p* = 0.59) or EPclin (15.1 ± 2.6% vs. 16.4 ± 2.8%; *p* = 0.55). There was a high degree of correlation between the W1 and W2% risk estimates for all signatures (RS; *r* = 0.93, ROR; *r* = 0.76, EP; *r* = 0.85 or EPclin; *r* = 0.83) (Supplementary Fig. 1b). The mean (± SEM) absolute difference in % risk estimates between W1 and W2 irrespective of direction of change was 3.6 ± 0.77% for RS, 2.2 ± 0.47% for ROR, 4.3 ± 0.92% for EP and 4.4 ± 0.93% for EPclin.

### Variation of scores measured in the same window vs. different windows

**Fig. 2:**
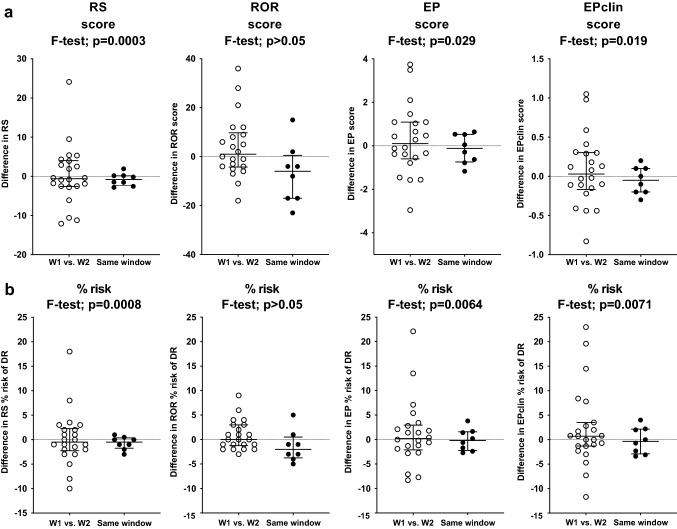
Comparison of changes in **a** RS, ROR, EP & EPclin scores and **b** % risk estimates of distant relapse, in paired tumour samples taken either in W1 (low oestrogen and progesterone) vs. W2 (high oestrogen ± progesterone) or in the same window

Measurements of the four signature scores in the same window, one menstrual cycle apart, from eight patients showed no significant changes (Fig. 2). The variation of the scores when they were measured in W1 and W2 compared to those measured in the same window was significantly higher for RS (F test; *p* = 0.0003) and EP/EPclin (*p* = 0.029 and 0.019, respectively), but not for ROR (*p* > 0.05) (Fig. 2a). Variation of the corresponding estimates of % risk of disease recurrence showed the same pattern with significant differences for RS (*p* = 0.0008) and EP/EPclin (*p* = 0.0064 and 0.0071, respectively), but again not for ROR (*p* > 0.05) (Fig. 2b).

### Changes in risk categories and intrinsic subtype classifications

Tumour samples were classified into their corresponding risk groups using the published cut-points for each signature [3–5]. For RS, ROR, EP and EPclin, 5 (23%), 6 (27%), 3 (14%) and 3 (14%), respectively, of the 22 tumours were assigned to a different risk category in W2 compared to W1 (Fig. 1a and Table 1). The kappa statistic (*κ*) measuring the agreement between the risk groups in the two windows was 0.66 (95% CI 0.40–0.92) for RS, 0.56 (95% CI 0.27–0.85) for ROR, 0.67 for EP (95% CI 0.34–1.00) and 0.73 for EPclin (95% CI 0.45–1.00). When measurements were made in the same window for RS, ROR, EP and EPclin, 0, 4 (50%), 3 (37%) and 1 (12%) of the 8 tumours were assigned to a different risk category, respectively. If the reduced cut-points for RS from the TAILORx study (intermediate group 11–25) [9] were used, 6 (27.3%) tumours were classified differently in W2 compared to W1 (*κ* = 0.54, 95% CI 0.27–0.80) and 4 (50%) tumours were classified differently when measured in the same window.

**Table 1:** Concordance of risk categorisation for paired measurements of (a) RS, (b) ROR, (c) EP and (d) EPclin scores performed in W1 (low oestrogen and progesterone) and W2 (high oestrogen ± progesterone) of the menstrual cycle

(a) RS					
RS	Risk category	W2			Total
		Low	Intermediate	High	
W1	Low	8	1	0	9
	Intermediate	1	4	1	6
	High	0	2	5	7
	Total	9	7	6	22

(b) ROR					
ROR	Risk category	W2			Total
		Low	Intermediate	High	
W1	Low	9	3	0	12
	Intermediate	1	4	1	6
	High	0	1	3	4
	Total	10	8	4	22

(c) EP				
EP	Risk category	W2		Total
		Low	High	
W1	Low	5	1	6
	High	2	14	16
	Total	7	15	22

(d) EPclin				
EPclin	Risk category	W2		Total
		Low	High	
W1	Low	8	3	11
	High	0	11	11
	Total	8	14	22

*RS* Oncotype® DX recurrence score, *ROR* Prosigna® PAM50 risk of recurrence score, *EP/EPclin* EndoPredict®, *W* window

The ROR test also provides intrinsic subtype information: 17 (77.3%) tumours were classified as Luminal A, 3 (13.6%) as Luminal B and one each as HER2-enriched (4.5%) and basal-like (4.5%) in W1. Three tumours (13.6%) had a different subtype classification in W2 compared to W1 (Luminal B to Luminal A, HER2-enriched to Luminal B & Luminal A to Luminal B). Two (25%) tumours had a different subtype assigned (both Luminal A to Luminal B) when measured in the same window.

### Changes in gene signature component modules and individual genes

**Fig. 3:**
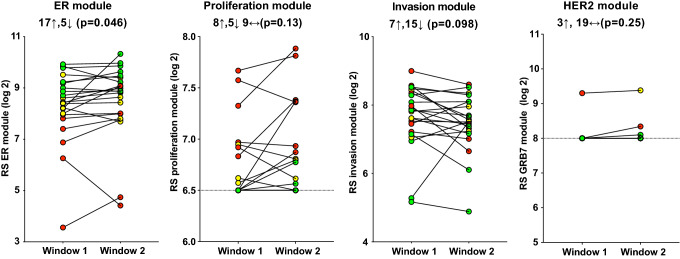
Changes in RS module scores in paired tumour samples taken in W1 (low oestrogen and progesterone) vs. W2 (high oestrogen ± progesterone). Tumours classified as low risk (RS < 18) are indicated in green, those at intermediate risk (RS 18–31) in yellow and those at high risk (RS > 31) in red

Of the individual modules of the RS, the mean ER module score was significantly higher in the window with high oestrogen (W2) (+ 16.6%; *p* = 0.046), whilst the mean invasion module score trended lower in W2 than W1 (− 10.9%; *p* = 0.098) with more than a twofold reduction in W2 in some patients (Fig. 3). The change in ER module score was driven by a significant increase in *PGR* expression between the two windows (+ 81.4%; *p* = 0.0029) with no change apparent in the other three genes (*ESR1*, *BCL2* and *SCUBE2*) in the module (Supplementary Fig. 2a). There was a trend for a higher RS proliferation module score (mean + 7.3%; *p* = 0.13) in W2, even though the score was thresholded in 13 cases in W1 and 10 cases in W2 (Fig. 3). All five of the individual PAGs that make up the RS proliferation module showed an increase in their mean expression in W2 compared to W1 (9.6–44.6%; *p* = 0.065–0.21) (Supplementary Fig. 2b), but in no case was this statistically significant. Both genes in the RS invasion module (*MMP11* and *CTSL2*) showed lower expression in W2, but this did not reach significance for either of them (Supplementary Fig. 2c). There was no significant change in the HER2 module scores, which were thresholded in 21/22 cases, between the windows (mean + 1.7%; *p* = 0.25) (Fig. 3).

The ROR proliferation score showed a non-significant trend to be higher in W2 compared to W1 (23.9%, *p* = 0.092; Supplementary Fig. 3) and there was a very strong correlation with the change in the ROR proliferation score and the change in ROR score between W1 and W2 (*r* = 0.86, *p* < 0.0001). Other than PGR (see above), no other individual gene in any of the signatures showed a significant change between W1 and W2.

### Correlation of RS, ROR and EPclin signature scores

RS, ROR and EPclin scores showed a stronger correlation with each other in W1 (RS vs. ROR: *r* = 0.69, *p* = 0.0004; ROR vs. EPclin: *r* = 0.81, *p* < 0.0001; RS vs. EPclin: *r* = 0.75, *p* =  < 0.0001) than in W2 (RS vs. ROR: *r* = 0.52, *p* = 0.014; ROR vs. EPclin: *r* = 0.65, *p* = 0.001; RS vs. EPclin: *r* = 0.70, *p* = 0.0003) (Supplementary Fig. 4). In both windows, RS and ROR showed the weakest correlation, whilst all correlations were stronger in W1 than W2.

**Fig. 4:**
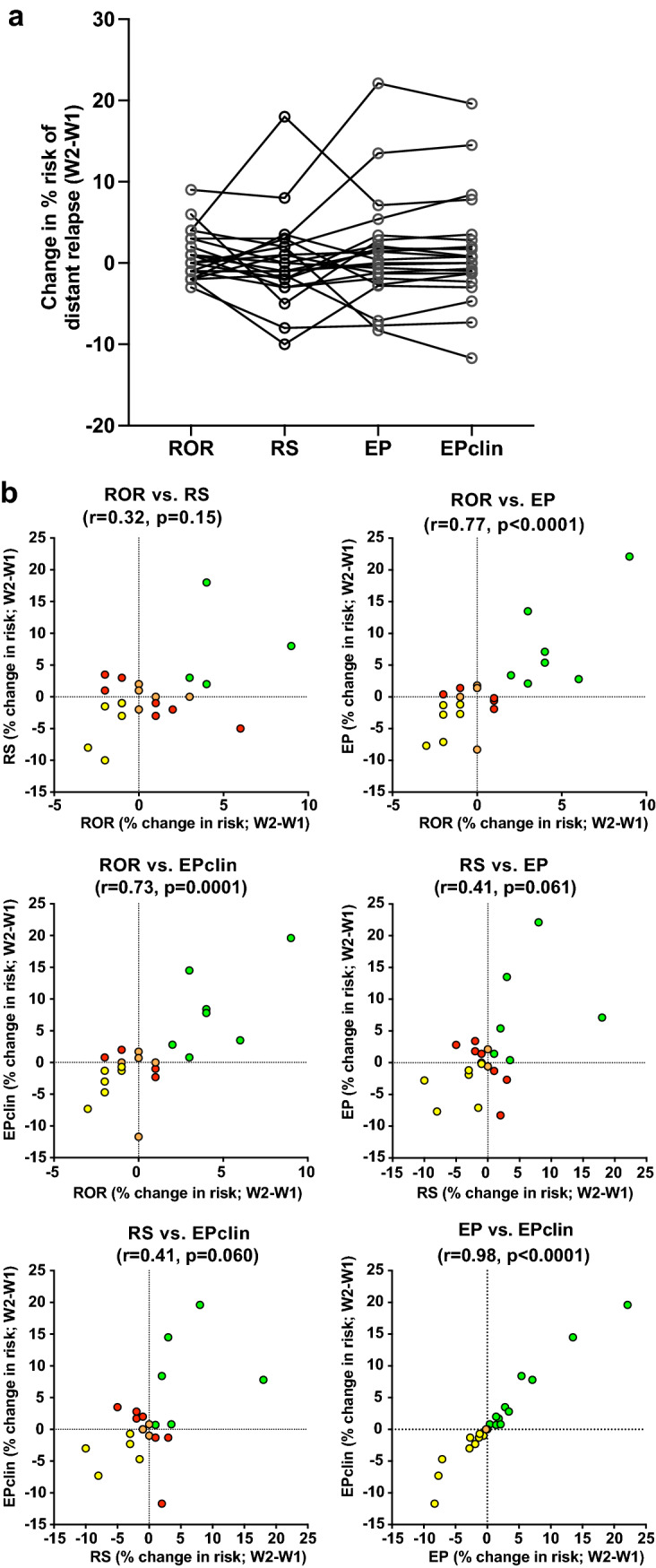
Change in % risk of distant relapse estimates between W1 (low oestrogen and progesterone) and W2 (high oestrogen ± progesterone) of the menstrual cycle for ROR, RS, EP and EPclin; **a** comparison and **b** correlation of changes. Concordant low-risk tumours indicated in green, concordant high-risk tumours in red and discordant risk tumours in orange (fully discordant) or yellow (no change in risk vs. low or high risk)

Changes in estimated risk between W1 and W2 with RS did not correlate significantly with the change in estimated risk with each of the other 3 signatures (range *r* = 0.32–0.41; *p* = 0.06–0.15). However, the change in estimated risk found in each of the other signatures did correlate significantly between each of those signatures (range *r* = 0.73–0.98; *p* ≤ 0.001), such that in most cases tumours showing an increase or decrease in risk with one test also showed an increase or decrease, respectively, with the other tests (Fig. 4).

## Discussion

Earlier studies examining changes in tumour biology during the menstrual cycle have focused mainly on ER and PgR protein levels and produced inconsistent results [20–27] reflecting the difficulties of reliably assigning the timing of the menstrual cycle. In more recent retrospective studies, we have shown tumoural ERG expression to be significantly higher in mid- to late cycle and PAG expression lower later in the cycle [13, 14]. In a prospective study, significant changes in the expression of ERGs and PAGs were demonstrated within the same tumour [15].

There is very little previous work examining the effect of menstrual cycle on gene expression-based prognostic signatures, such as RS, ROR and EP, which are widely used in ER-positive breast cancer to estimate the risk of distant recurrence for patients receiving endocrine therapy and help guide the use of adjuvant chemotherapy. A recent study by Bernhardt et al. [28] in 25 women reported a higher discordance of RS score when measured in paired samples from the 16 women < 50 years of age. Eight of the 16 cases < 50 years showed differences of > 4 U in the recurrence score between the paired biopsy compared with none of the 9 cases from older women. The calculation of an ‘analogous’ RS in that study did not appear to threshold the proliferation and HER2 modules as performed in the clinically used RS algorithm so the results may not correctly replicate the clinically used RS. This observation highlights the importance of using methodology able to accurately recapitulate clinical prognostic signature scores in the research setting. In the current study, we used our published method for the derivation of RUO RS and EP/EPclin scores using gene expression data generated on the NanoString nCounter platform [18]. Nonetheless, the data from the Bernhardt study support the concept of substantially greater variation in RSs in premenopausal than in postmenopausal women.

Although none of the individual gene signatures showed systematic changes in their score or their estimate of risk of distant recurrence in the absence of chemotherapy between the different windows of the menstrual cycle, substantial variability was observed between paired samples for all three scores. Some of these changes might result in different clinical decision-making regarding the use of chemotherapy in the affected patient. However, it is not possible to say whether results might be more accurately aligned to clinical outcome if tests were conducted in one window rather than the other. The lower % discordance for EP/EPclin would be expected due to the absence of an intermediate risk group for these scores and therefore less potential for discordance. There were also risk categorisation changes in the small group of control samples taken within the same window of the menstrual cycle suggesting that a significant proportion of the variation observed may be inherent to the assays, tissue heterogeneity or subtle menstrual cycle effects.

The proportion of patients that switch from one category to another is clinically relevant and is most easily judged with the EPclin where there are just low- and high-risk categories. In this study set 3/22 (14%) differed in this way but the size of the study does not allow this to be considered as generalisable. The proportion switching will also vary according to the population in which this is assessed with higher proportions occurring when estimates are close to the risk category cut-off.

It should be noted that changes in risk categorisation can give a very variable read-out of a test’s reproducibility. Thus, when the revised cut-points (11–25) for RS from the TAILORx study [9] were used, 50% of tumours were classified differently in the same window, whereas there were no misclassifications using the original cut-off values. The changes seen in the intrinsic subtype information provided by the ROR test were similar between samples taken in different windows (14%) and samples taken in the same window (25%) providing no evidence for any additional variation in intrinsic subtype determination due to menstrual cycle effects.

Comparison of the variation of the signature scores and their estimates of % risk of distant recurrence in the absence of chemotherapy when they were measured in W1 and W2 compared to in the same window indicated a significant difference for RS and EP/EPclin, but not for ROR suggesting a greater effect of the menstrual cycle on the former signatures with the caveat that the numbers for comparison are low in the same window group. Alternatively, this may reflect a greater inherent variability in the ROR score, such that it is harder to detect a difference in variability between the pairs of measurements in the same and the different windows. Published analytical and reproducibility data for the clinical versions of the tests show standard deviations of 1.53 (1.53% of reporting range) for RS [29], 0.21 (1.40% of reporting range) for EP, 0.057 (0.70% of reporting range) for EPclin [30] and 2.9 (2.9% of reporting range) for ROR [31], with a 90% concordance of subtype classifications for the latter. This provides some evidence for a greater inherent variability of the ROR score, although the data underlying these estimates come from different populations and the comparisons are therefore indirect. Interestingly, RS, ROR and EPclin scores showed stronger correlations with each other in W1 than in W2 possibly reflecting the less variable hormonal milieu in W1. Incorporation of clinical information might be expected to reduce the observed variability between paired measurements in the same patient as it is identical for both sample pairs. However, there was little evidence for this when EPclin was compared to EP.

The variability of the RS during the menstrual cycle was investigated further by examining changes in its component modules and genes. The ER module score was significantly higher in the presence of the higher oestrogen and progesterone levels in W2 rather than in W1, driven by a significant increase in *PGR* expression. Additionally, the proliferation module score, even though thresholded, showed a trend to increase in W2, whilst the invasion module score trended lower in W2. These data confirm that changes in individual genes and gene modules do occur across the cycle, but that these changes largely balance one another out because of their opposite direction in the risk algorithm for the RS. In agreement with the trend for the RS proliferation score to increase in W2, the ROR proliferation score also showed a strong trend to increase in W2. The change in ROR proliferation score correlated very strongly with the change in ROR between windows. This concurs with recent work indicating that proliferation appears to be the main driver of ROR, in contrast to RS, which may be more driven by its ER module (and predominantly by PGR itself) in a postmenopausal population [8].

Strengths of the current study include the careful assignment of menstrual cycle timing, the use of validated methodology to accurately recapitulate the prognostic signature scores and the availability of a group of tumours taken in the same window to act as a control. A weakness of the study was the modest number of patients available particularly for those pairs of samples taken in the same window. To maximise numbers, we used samples from two independent studies [15] and split the menstrual cycle into just two windows. As a consequence, W2 contained a wide range of progesterone concentrations in particular, ranging from very low in the first half of W2 to maximal in the latter half of the window. This would be likely to add extra variability to measurements made in W2, thereby reducing the power of the study to observe significant differences between paired samples taken in W1 and W2. Another limitation of the study is the inclusion of patients with node positivity although the RxPONDER trial found no evidence that OncotypeDX is informative for choosing whether patients should receive chemotherapy. There is no reason to expect that variability in molecular scores of the primary will vary according to lymph node status but this would impact on the estimates of risk of distant recurrence.

In summary, we show that there are significant changes during the menstrual cycle in the expression of some of the genes and gene module scores comprising the RS, ROR and EP/EPclin scores, but these do not affect any of the prognostic scores in a systematic fashion. Whilst none of the individual gene signatures showed significant changes between different windows of the menstrual cycle, substantial variability was observed for all three scores, such that 14–27% of samples were assigned to a different risk category.

## Supplementary Information

Below is the link to the electronic supplementary material.

Supplementary file1 (PDF 316 kb)

Supplementary file2 (PDF 115 kb)

## Data Availability

The datasets generated during and/or analysed during the current study are available from the corresponding author on reasonable request.

## Abbreviations

EP/EPclin: EndoPredict®
ER: Oestrogen receptor
ERG: Oestrogen-regulated gene
HER2: Human epidermal growth factor receptor-2
PAG: Proliferation-associated gene
PgR: Progesterone receptor
ROR: Prosigna® PAM50 risk of recurrence score
RS: Oncotype® DX recurrence score
RSPC: Recurrence score-pathology-clinical
RUO: Research use only
SEM: Standard error of the mean
