# Supplementary material for: Impact of the menstrual cycle on commercial prognostic gene signatures in oestrogen receptor-positive primary breast cancer
Source: Breast Cancer Res Treat. 2021 Sep 15;190(2):295–305. doi: 10.1007/s10549-021-06377-3 (PMC8558287; doi:10.1007/s10549-021-06377-3)
Supplement: Supplementary file 1 — Supplementary file1 (PDF 316 kb) [file 10549_2021_6377_MOESM1_ESM.pdf]

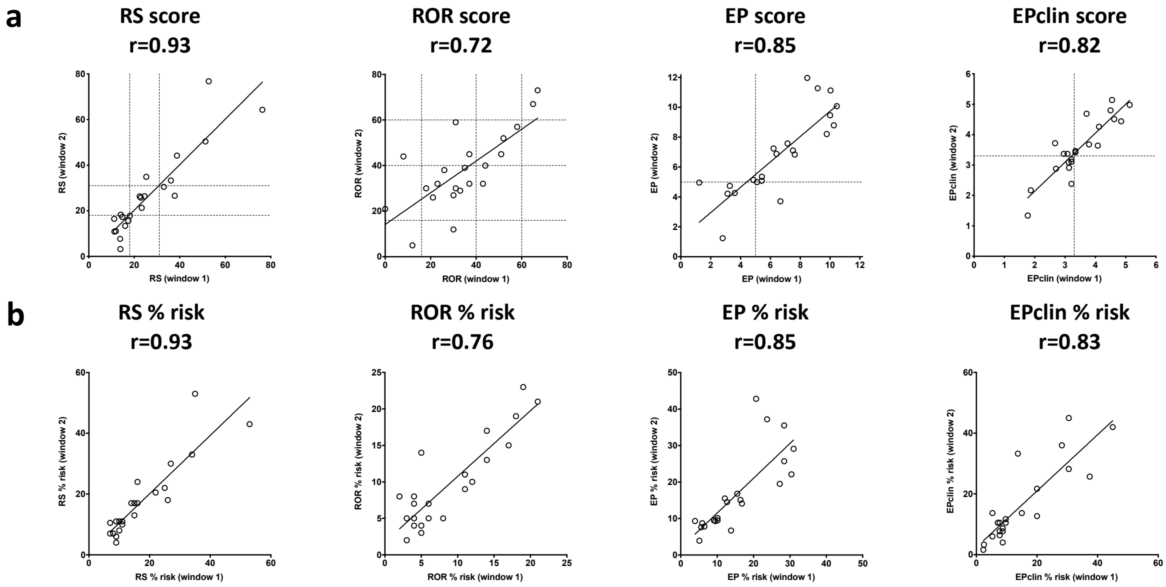

Supplementary figure 1 Correlation of (a) RS, ROR, EP & EPclin scores and (b) % risk estimates of distant relapse, in paired tumour samples taken in W1 (low oestrogen and progesterone) vs W2 (high oestrogen ± progesterone).

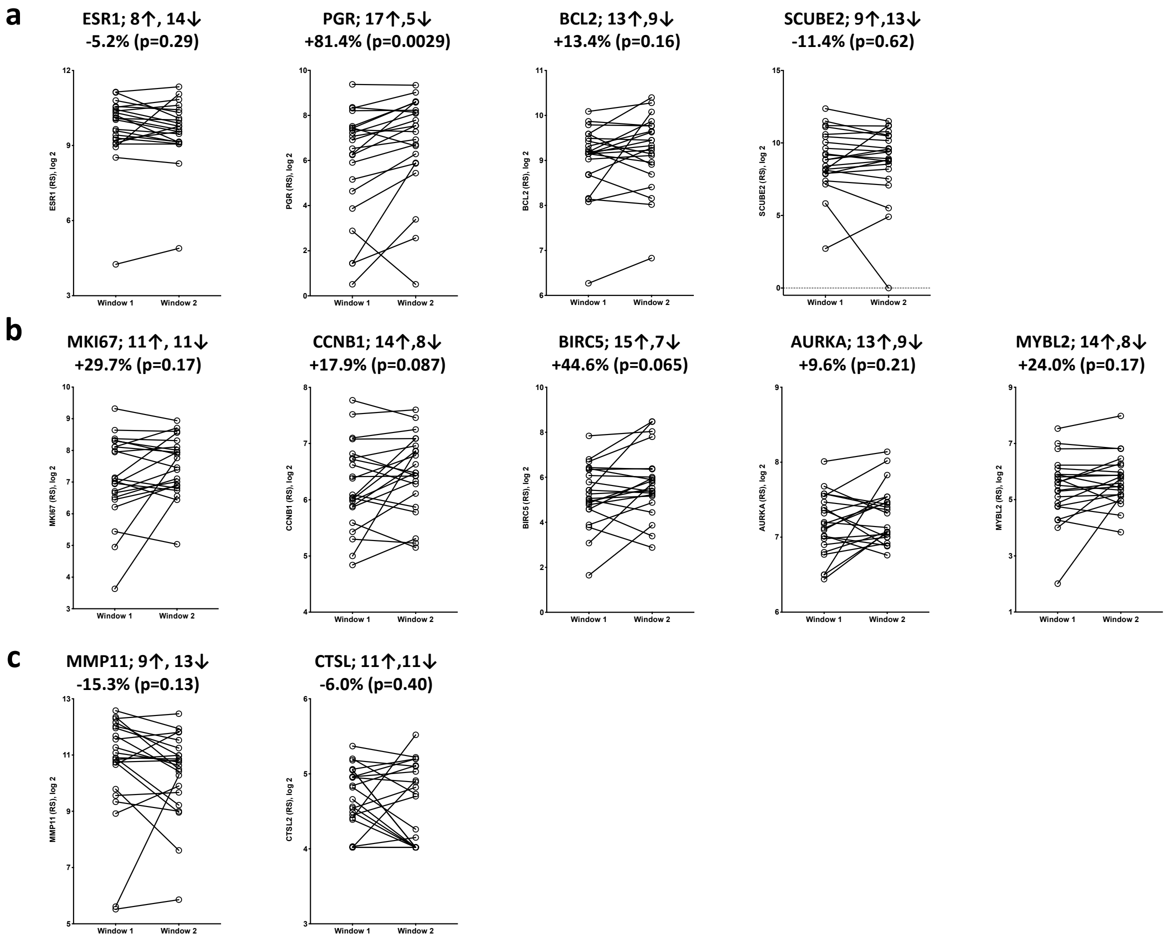

Supplementary Fig. 2 Change in expression of RS module genes between W1 (low oestrogen and progesterone) vs W2 (high oestrogen ± progesterone): (a) ER module (b) proliferation module and (c) invasion module.

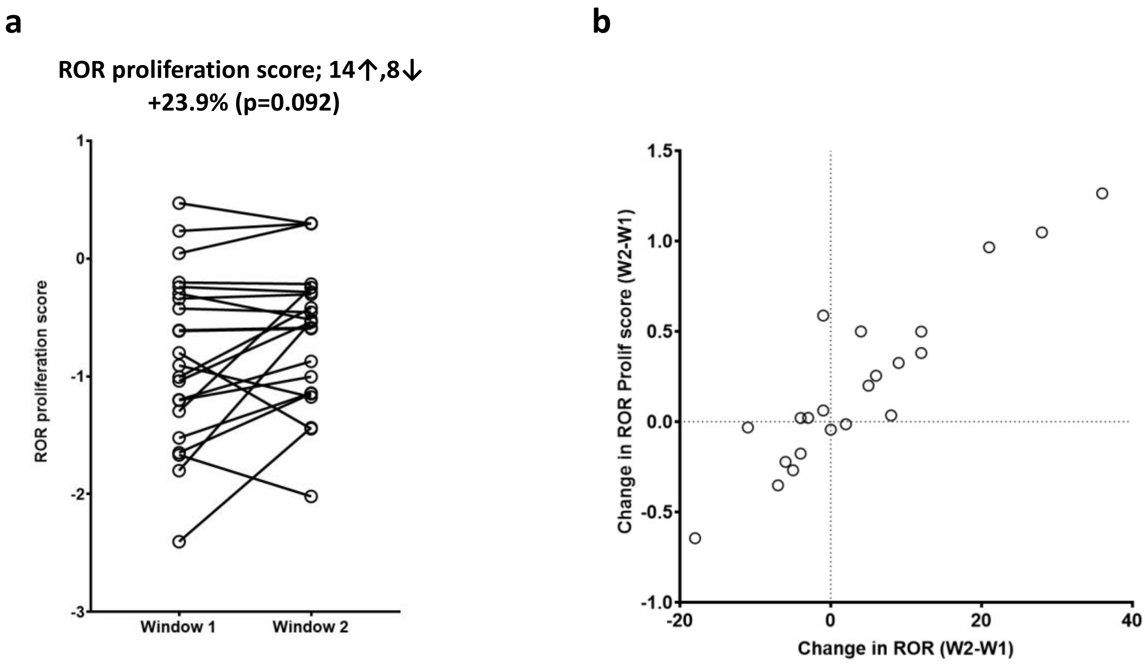

Supplementary Fig. 3 Change in (a) ROR proliferation score and (b) correlation of change in proliferation score with change in ROR score, in paired tumour samples taken in W1 (low oestrogen and progesterone) vs W2 (high oestrogen ± progesterone).

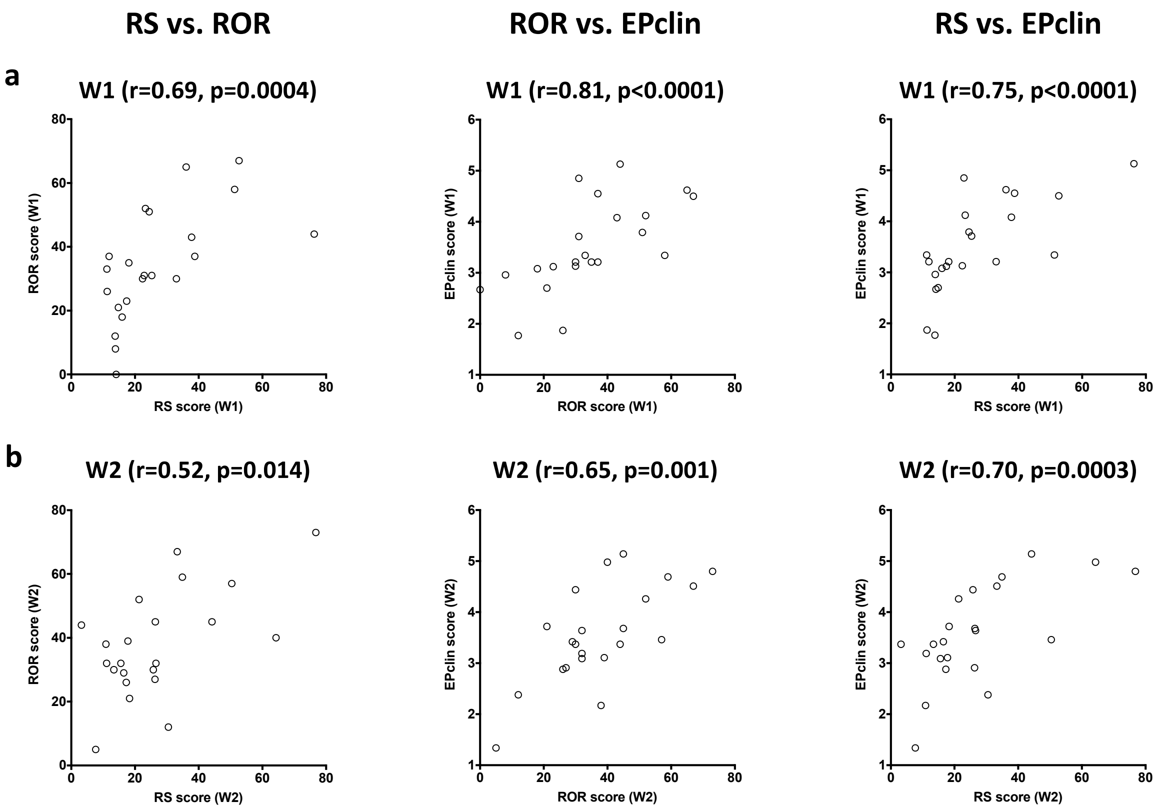

Supplementary Fig. 4 Correlation of RS, ROR and EPclin scores in samples taken in (a) W1 (low oestrogen and progesterone) and (b) W2 (high oestrogen ± progesterone).
