# Supplementary material for: Impact of the menstrual cycle on commercial prognostic gene signatures in oestrogen receptor-positive primary breast cancer
Source: Breast Cancer Res Treat. 2021 Sep 15;190(2):295–305. doi: 10.1007/s10549-021-06377-3 (PMC8558287; doi:10.1007/s10549-021-06377-3)
Supplement: Supplementary file 2 — Supplementary file2 (PDF 115 kb) [file 10549_2021_6377_MOESM2_ESM.pdf]

Supplementary table 1. The custom NanoString gene expression panel used in the study.

HUGO Gene	Accession	NSID	Target Sequence	Reference
ACTB	NM_001101.2	NM_001101.2:1685	CCAACTTGAGATGTATGAAGGCTTTTGGTCTCCCTGGGAGTGGGTG GAGGCAGCCAGGGCTTACCTGTACACTGACTTGAGACCAGTTGAAT AAAAGTGC	HK
ACTR3B	NM_001040135.1	NM_001040135.1:905	CCAGAAGAAGTTTGTTATAGACGTTGGTTACGAAAGATTCCTGGGA CCTGAAATATTCTTTCACCCGGAGTTTGCCAACCCAGACTTTATGGA GTCCATC	
ANLN	NM_018685.2	NM_018685.2:240	CGTGCCAGGCGAGAGAATCTTCAGAGAAAAATGGCTGAGAGGCCC ACAGCAGCTCCAAGGTCTATGACTCATGCTAAGCGAGCTAGACAGC CACTTTCAG	
AURKA	NM_003600.2	NM_003600.2:405	AGCTCCAGTTGGAGGTCCAAAACGTGTTCTCGTGACTCAGCAATTT CCTTGTCAGAATCCATTACCTGTAAATAGTGGCCAGGCTCAGCGGG TCTTGTGT	
AZGP1	NM_001185.2	NM_001185.2:123	GTTACTCTCTGACCTATATCTACACTGGGCTGTCCAAGCATGTTGAA GACGTCCCCGCGTTTCAGGCCCTTGGCTCACTCAATGACCTCCAGT TCTTTAG	
BAG1	NM_004323.3	NM_004323.3:540	CTTCATGTTACCTCCCAGCAGGGCAGCAGTGAACCAGTTGTCCAAG ACCTGGCCCAGGTTGTTGAAGAGGTCATAGGGGTTCCACAGTCTTT TCAGAAAC	
BCL2	NM_000633.2	NM_000633.2:1525	CCAAGCACCGCTTCGTGTGGCTCCACCTGGATGTTCTGTGCCTGTA AACATAGATTCGCTTTCCATGTTGTTGGCCGGATCACCATCTGAAGA GCAGACG	
BIRC5	NM_001168.2	NM_001168.2:1215	CCATTCTAAGTCATTGGGGAAACGGGGTGAACTTCAGGTGGATGAG GAGACAGAATAGAGTGATAGGAAGCGTCTGGCAGATACTCCTTTTG CCACTGCT	
BLVRA	NM_000712.3	NM_000712.3:485	TTCCTGAAAAAAGAAGTGGTGGGGAAAGACCTGCTGAAAGGGTCG CTCCTCTTCACAGCTGGCCCGTTGGAAGAAGAGCGGTTTGGCTTCC CTGCATTCA	
BUB1B	NM_001211.4	NM_001211.4:835	GAGTCTTCTGTACCACAACGAAGCACACTAGCTGAACTAAAGAGCA AAGGGAAAAAGACAGCAAGAGCTCCAATCATCCGTGTAGGAGGTG CTCTCAAGG	

CALM2	NM_001743.3	NM_001743.3:868	TGGAGTTGTAACTCTGCGTGGACTATGGACAGTCAACAATATGTACTTAAAAGTTGCACTATTGCAAAACGGGTGTATTATCCAGGTACTCGTACACTAT	HK
CCNB1	NM_031966.2	NM_031966.2:710	GAGACAACTTGAGGAAGAGCAAGCAGTCAGACCAAAATACCTACTGGGTCGGGAAGTCACTGGAAACATGAGAGCCATCCTAATTGACTGGCTAGTACAG	
CCNE1	NM_001238.1	NM_001238.1:1155	GAGAACTGTGTCAAGTGGATGGTTCCATTTGCCATGGTTATAAGGGAGACGGGGAGCTCAAAACTGAAGCACTTCAGGGGCGTCGCTGATGAAGATGCAC	
CD68	NM_001251.2	NM_001251.2:1140	ACCGGTCCATCTTGCTGCCTCTCATCATCGGCCTGATCCTTCTTGGCCTCCTCGCCCTGGTGCTTATTGCTTTCTGCATCATCCGGAGACGCCCATCCGC	
CDC20	NM_001255.1	NM_001255.1:915	CCCGAGTGGGCTCCCTAAGCTGGAACAGCTATATCCTGTCCAGTGGTTCACGTTCTGGCCACATCCACCACCATGATGTTCGGGTAGCAGAACACCATGT	
CDC6	NM_001254.3	NM_001254.3:1655	GGGGAAGTTATATGAAGCCTACAGTAAAGTCTGTCGCAAACAGCAGGTGGCGGCTGTGGACCAGTCAGAGTGTTTGTCACTTTCAGGGCTCTTGGAAGCC	
CDH3	NM_001793.3	NM_001793.3:2005	CCCTCGACCGTGAGGATGAGCAGTTTGTGAGGAACAACATCTATGAAGTCATGGTCTTGGCCATGGACAATGGAAGCCCTCCCACCACTGGCACGGGAAC	
CENPA	NM_001042426.1	NM_001042426.1:979	CACTTTGAGCAGTTGCCTGGAAGGCTGGGCATTTCCATCATATAGACCTCTGCCCTTCAGAGTAGCCTCACCATTAGTGGCAGCATCATGTAACTGAGTG	
CENPF	NM_016343.3	NM_016343.3:9260	AGAAAATCTTGCAGAGTCCTCCAAACCAACAGCTGGTGGCAGCAGATCACAAAAGGTCAAAGTTGCTCAGCGGAGCCCAGTAGATTCAGGCACCATCCTC	
CEP55	NM_018131.3	NM_018131.3:570	GTACTACCGCATTGCTTGAACAGCTGGAAGAGACAACGAGAGAAGGAGAAAGGAGGGAGCAGGTGTTGAAAGCCTTATCTGAAGAGAAAGACGTATTGAA	
CTSV	NM_001333.3	NM_001333.3:2820	GTGAAATTTAATCGAAAGGTGATCCATTGTGAATGCAATGGGAGGGAAGGGGCATGTGGGACTGTGTATCCCAAAAACCCTTTGATAGCCTATGTCCACA	

CXXC5	NM_016463.5	NM_016463.5:1630	AGCTGCCCTCTCCGTGCAATGTCACTGCTCGTGTGGTCTCCAGCAAGGGATTCGGGCGAAGACAAACGGATGCACCCGTCTTTAGAACCAAAAATATTCT	
DHCR7	NM_001360.2	NM_001360.2:780	CCCACCATCATCTTCGACAACTGGATCCCACTGCTGTGGTGCGCCAACATCCTTGGCTATGCCGTCTCCACCTTCGCCATGGTCAAGGGCTACTTCTTCC	
EGFR	NM_005228.3	NM_005228.3:2760	GCAGCCAGGAACGTACTGGTGAAAACACCGCAGCATGTCAAGATCACAGATTTTGGGCTGGCCAAACTGCTGGGTGCGGAAGAGAAAGAATACCATGCAG	
ERBB2	NM_004448.2	NM_004448.2:2405	TGAAGGTGCTTGGATCTGGCGCTTTTGGCACAGTCTACAAGGGCATCTGGATCCCTGATGGGGAGAATGTGAAAATTCCAGTGGCCATCAAAGTGTTGAG	
ESR1	NM_000125.2	NM_000125.2:1595	AGGAACCAGGGAAAATGTGTAGAGGGCATGGTGGAGATCTTCGACATGCTGCTGGCTACATCATCTCGGTTCCGCATGATGAATCTGCAGGGAGAGGAGT	
EXO1	NM_006027.3	NM_006027.3:820	TGGCCCACAAAGTAATTAAAGCTGCCCGGTCTCAGGGGGTAGATTGCCTCGTGGCTCCCTATGAAGCTGATGCGCAGTTGGCCTATCTTAACAAAGCGGG	
FGFR4	NM_002011.3	NM_002011.3:1002	CCCACATCCAGTGGCTGAAGCACATCGTCATCAACGGCAGCAGCTTCGGAGCCGACGGTTTCCCCTATGTGCAAGTCCTAAAGACTGCAGACATCAATAG	
FOXA1	NM_004496.2	NM_004496.2:280	TGGATGGTTGTATTGGGCAGGGTGGCTCCAGGATGTTAGGAACTGTGAAGATGGAAGGGCATGAAACCAGCGACTGGAACAGCTACTACGCAGACACGCA	
FOXC1	NM_001453.1	NM_001453.1:1530	TTCGAGTCACAGAGGATCGGCTTGAACAACTCTCCAGTGAACGGGAATAGTAGCTGTCAAATGGCCTTCCCTTCCAGCCAGTCTCTGTACCGCACGTCCG	
GAPDH	NM_002046.3	NM_002046.3:972	CACTCCTCCACCTTTGACGCTGGGGCTGGCATTGCCCTCAACGACCACTTTGTCAAGCTCATTTCCTGGTATGACAACGAATTTGGCTACAGCAACAGGG	HK
GPR160	NM_014373.1	NM_014373.1:760	GGATTTCAGTCCTTGCTTATGTTTTGGGAGACCCAGCCATCTACCAAAGCCTGAAGGCACAGAATGCTTATTCTCGTCACTGTCCTTTCTATGTCAGCAT	

GRB7	NM_005310.2	NM_005310.2:1010	GCCGATCTGGCCTCTATTACTCCACCAAGGGCACCTCTAAGGATCCGAGGCACCTGCAGTACGTGGCAGATGTGAACGAGTCCAACGTGTACGTGGTGAC	
GSTM1	NM_000561.2	NM_000561.2:335	GATTCGTGTGGACATTTTGGAGAACCAGACCATGGACAACCATATGCAGCTGGGCATGATCTGCTACAATCCAGAATTTGAGAAACTGAAGCCAAAGTAC	
GUSB	NM_000181.1	NM_000181.1:1350	CGGTCGTGATGTGGTCTGTGGCCAACGAGCCTGCGTCCCACCTAGAATCTGCTGGCTACTACTTGAAGATGGTGATCGCTCACACCAAATCCTTGGACCC	HK
HMBS	NM_000190.3	NM_000190.3:315	CATTGCTATGTCCACCACAGGGGACAAGATTCTTGATACTGCACTCTCTAAGATTGGAGAGAAAAGCCTGTTTACCAAGGAGCTTGAACATGCCCTGGAG	HK
HOXB13	NM_006361.5	NM_006361.5:1220	CCACCAGGGTTCCCAAAGAACCTGGCCCAGTCATAATCATTCATCCTGACAGTGGCAATAATCACGATAACCAGTACTAGCTGCCATGATCGTTAGCCTC	
IL17RB	NM_018725.3	NM_018725.3:688	TCGGGTTTTCTCAGGTGTTTGAGCCACACCAGAAGAAACAAACGCGAGCTTCAGTGGTGATTCCAGTGACTGGGGATAGTGAAGGTGCTACGGTGCAGCT	
IL6ST	NM_002184.2	NM_002184.2:2505	CAAAACACTTCGAGCACTGTCCAGTATTCTACCGTGGTACACAGTGGCTACAGACACCAAGTTCCGTCAGTCCAAGTCTTCTCAAGATCCGAGTCTACCC	
KIF2C	NM_006845.2	NM_006845.2:1020	GTTGTCTACAGGTTCACAGCAAGGCCACTGGTACAGACAATCTTTGAAGGTGGAAAAGCAACTTGTTTTGCATATGGCCAGACAGGAAGTGGCAAGACAC	
KRT14	NM_000526.3	NM_000526.3:1365	GCAGTCATCCAGAGATGTGACCTCCTCCAGCCGCCAAATCCGCACCAAGGTCATGGATGTGCACGATGGCAAGGTGGTGTCCACCCACGAGCAGGTCCTT	
KRT17	NM_000422.1	NM_000422.1:1230	CTGACTCAGTACAAGAAAGAACCGGTGACCACCCGTCAGGTGCGTACCATTGTGGAAGAGGTCCAGGATGGCAAGGTCATCTCCTCCCGCGAGCAGGTCC	
KRT5	NM_000424.2	NM_000424.2:130	CTGGTTCTCTTGCTCCACCAGGAACAAGCCACCATGTCTCGCCAGTCAAGTGTGTCCTTCCGGAGCGGGGGCAGTCGTAGCTTCAGCACCGCCTCTGCCA	

MAPT	NM_016835.3	NM_016835.3:1425	GCCGGGTCCCTCAACTCAAAGCTCGCATGGTCAGTAAAAGCAAAGACGGGACTGGAAGCGATGACAAAAAAGCCAAGACATCCACACGTTCCTCTGCTAA	
MDM2	NM_006878.2	NM_006878.2:280	GGTGAGGAGCAGGCAAATGTGCAATACCAACATGTCTGTACCTACTGATGGTGCTGTAACCACCTCACAGATTCCAGCTTCGGAACAAGAGACCCTGGTT	
MELK	NM_014791.2	NM_014791.2:365	AGAGACAGCCAACAAAATATTCATGGTTCTTGAGTACTGCCCTGGAGGAGAGCTGTTTGACTATATAATTTCCCAGGATCGCCTGTCAGAAGAGGAGACC	
MGP	NM_000900.2	NM_000900.2:305	TCAATAGGGAAGCCTGTGATGACTACAGACTTTGCGAACGCTACGCCATGGTTTATGGATACAATGCTGCCTATAATCGCTACTTCAGGAAGCGCCGAGG	
MIA	NM_006533.1	NM_006533.1:265	CCGGGGCCAAGTGGTGTATGTCTTCTCCAAGCTGAAGGGCCGTGGGCGGCTCTTCTGGGGAGGCAGCGTTCAGGGAGATTACTATGGAGATCTGGCTGCT	
MKI67	NM_002417.2	NM_002417.2:2005	GCTTCCAGCAGCAAATCTCAGACAGAGGTTCCTAAGAGAGGAGGAGAAAGAGTGGCAACCTGCCTTCAAAAGAGAGTGTCTATCAGCCGAAGTCAACATG	
MLPH	NM_024101.4	NM_024101.4:1695	GAGGAAGTCAAACCTCCCGATATTTCTCCCTCGAGTGGCTGGGAAACTTGGCAAGAGACCAGAGGACCCAAATGCAGACCCTTCAAGTGAGGCCAAGGCA	
MMP11	NM_005940.3	NM_005940.3:702	AGCAGCCAAGGCCCTGATGTCCGCCTTCTACACCTTTCGCTACCCACTGAGTCTCAGCCCAGATGACTGCAGGGGCGTTCAACACCTATATGGCCAGCCC	
MRPL19	NM_014763.3	NM_014763.3:385	ACAGCTGACCCATATGCCAGTGGAAAAATCAGCCAGTTTCTGGGGATTTGCATTCAGAGATCAGGAAGAGGACTTGGAGCTACTTTCATCCTTAGGAATG	HK
MYBL2	NM_002466.2	NM_002466.2:675	GCAACCGCTGGGCCGAGATCGCCAAGATGTTGCCAGGGAGGACAGACAATGCTGTGAAGAATCACTGGAACTCTACCATCAAAAGGAAGGTGGACACAGG	
MYC	NM_002467.3	NM_002467.3:1615	CACCGAGGAGAATGTCAAGAGGCGAACACACAACGTCTTGGAGCGCCAGAGGAGGAACGAGCTAAAACGGAGCTTTTTTGCCCTGCGTGACCAGATCCCG	

NAT1	NM_000662.4	NM_000662.4:0	AGCACTTCCTCATAGACCTTGGATGTGGGAGGATTGCATTCAGTCTAGTTCCTGGTTGCCGGCTGAAATAACCTGAATTCAAGCCAGGAAGAAGCAGCAA	
NDC80	NM_006101.1	NM_006101.1:90	AAAAGGTCATAAGCATGAAGCGCAGTTCAGTTTCCAGCGGTGGTGCTGGCCGCCTCTCCATGCAGGAGTTAAGATCCCAGGATGTAAATAAACAAGGCCT	
NEK2	NM_002497.3	NM_002497.3:480	AGAGTTTGTTCTTCGAGTGATGACTCAGTTGACTCTGGCCCTGAAGGAATGCCACAGACGAAGTGATGGTGGTCATACCGTATTGCATCGGGATCTGAAA	
NUF2	NM_145697.1	NM_145697.1:215	GCCTGGCGGTGTTTTCGTCGTGCTCAGCGGTGGGAGGAGGCGGAAGAAACCAGAGCCTGGGAGATTAACAGGAAACTTCCAAGATGGAAACTTTGTCTTT	
OAZ1	NM_004152.2	NM_004152.2:313	GGTGGGCGAGGGAATAGTCAGAGGGATCACAATCTTTCAGCTAACTTATTCTACTCCGATGATCGGCTGAATGTAACAGAGGAACTAACGTCCAACGACA	HK
ORC6	NM_014321.2	NM_014321.2:580	GACTGTGTAAACAACTAGAGAAGATTGGACAGCAGGTCGACAGAGAACCTGGAGATGTAGCTACTCCACCACGGAAGAGAAAGAAGATAGTGGTTGAAGC	
PGR	NM_000926.2	NM_000926.2:3165	GGGATGAAGCATCAGGCTGTCATTATGGTGTCCTTACCTGTGGGAGCTGTAAGGTCTTCTTTAAGAGGGCAATGGAAGGGCAGCACAACTACTTATGTGC	
PHGDH	NM_006623.2	NM_006623.2:505	GCGACGGCTTCGATGAAGGACGGCAAATGGGAGCGGAAGAAGTTCATGGGAACAGAGCTGAATGGAAAGACCCTGGGAATTCTTGGCCTGGGCAGGATTG	
PSMC4	NM_006503.2	NM_006503.2:300	CATCGGACAATTTCTGGAGGCTGTGGATCAGAATACAGCCATCGTGGGCTCTACCACAGGCTCCAACTATTATGTGCGCATCCTGAGCACCATCGATCGG	HK
PTTG1	NM_004219.2	NM_004219.2:202	CACCAGCCTTACCTAAAGCTACTAGAAAGGCTTTGGGAACTGTCAACAGAGCTACAGAAAAGTCTGTAAAGACCAAGGGACCCCTCAAACAAAAACAGCC	
PUM1	NM_001020658.1	NM_001020658.1:640	CTGGGGAACATCAGATCATTCAGTTTCCCAGCCAATCATGGTGCAGAGAAGACCTGGTCAGAGTTTCCATGTGAACAGTGAGGTCAATTCTGTACTGTCC	

RACGAP1	NM_013277.3	NM_013277.3:1850	CAGTGACAATGTTACAGGACATCAAGCGTCAACCCAAGGTGGTTGAGCGCCTGCTTTCCTTGCCTCTGGAGTATTGGAGTCAGTTCATGATGGTGGAGCA	
RBBP8	NM_002894.2	NM_002894.2:760	AATGATCAACAGCATCAAGCAGCTGAGCTTGAATGTGAGGAAGACGTTATTCCAGATTCACCGATAACAGCCTTCTCATTTTCTGGCGTTAACCGGCTAC	
RPL37A	NM_000998.4	NM_000998.4:298	CTTCCGCTGTCACGGTAAAGTCCGCCATCAGAAGACTGAAGGAGTTGAAAGACCAGTAGACGCTCCTCTACTCTTTGAGACATCACTGGCCTATAATAAA	HK
RPLP0	NM_001002.3	NM_001002.3:250	CGAAATGTTTCATTGTGGGAGCAGACAATGTGGGCTCCAAGCAGATGCAGCAGATCCGCATGTCCCTTCGCGGGAAGGCTGTGGTGCTGATGGGCAAGAA	HK
RRM2	NM_001034.1	NM_001034.1:490	TTCCTTTTGGACCGCCGAGGAGGTTGACCTCTCCAAGGACATTCAGCACTGGGAATCCCTGAAACCCGAGGAGAGATATTTTATATCCCATGTTCTGGCT	
SCUBE2	NM_020974.1	NM_020974.1:1835	CGTAAAGCCATCCGCACGCTCAGAAAGGCCGTCCACAGGGAGCAGTTTCACCTCCAGCTCTCAGGCATGAACCTCGACGTGGCTAAAAAGCCTCCCAGAA	
SDHA	NM_004168.1	NM_004168.1:230	TGGAGGGGCAGGCTTGCGAGCTGCATTTGGCCTTTCTGAGGCAGGGTTTAATACAGCATGTGTTACCAAGCTGTTTCCTACCAGGTCACACACTGTTGCA	HK
SF3A1	NM_005877.4	NM_005877.4:1485	GATGATGAGGTGTACGCACCAGGTCTGGATATTGAGAGCAGCTTGAAGCAGTTGGCTGAGCGGCGTACTGACATCTTCGGTGTAGAGGAAACAGCCATTG	HK
SFRP1	NM_003012.3	NM_003012.3:1320	GTGGGTCACACACACGCACTGCGCCTGTCAGTAGTGGACATTGTAATCCAGTCGGCTTGTTCTTGCAGCATTCCCGCTCCCTTCCCTCCATAGCCACGCT	
SLC39A6	NM_012319.2	NM_012319.2:1580	GATCGAACTGAAGGCTATTTACGAGCAGACTCACAAGAGCCCTCCCACTTTGATTCTCAGCAGCCTGCAGTCTTGGAAGAAGAAGAGGTCATGATAGCTC	
STC2	NM_003714.2	NM_003714.2:2825	ATTTCTATGTGTAATTTCTGAGCCATTGTACTGTCTGGGCTGGGGGGGACACTGTCCAAGGGAGTGGCCCCTATGAGTTTATATTTTAACCACTGCTTCA	

TFRC	NM_003234.1	NM_003234.1:1220	CAGTTTCCACCATCTCGGTCATCAGGATTGCCTAATATACCTGTCCAGACAATCTCCAGAGCTGCTGCAGAAAAGCTGTTTGGGAATATGGAAGGAGACT	HK
TMEM45B	NM_138788.3	NM_138788.3:730	CTGGCTGCCCTCAGCATTGTGGCCGTCAACTATTCTCTTGTTTACTGCCTTTTGACTCGGATGAAGAGACACGGAAGGGGAGAAATCATTGGAATTCAGA	
TYMS	NM_001071.1	NM_001071.1:395	TGCTAAAGAGCTGTCTTCCAAGGGAGTGAAAATCTGGGATGCCAATGGATCCCGAGACTTTTTGGACAGCCTGGGATTCTCCACCAGAGAAGAAGGGGAC	
UBC	NM_021009.3	NM_021009.3:1875	TGCAGATCTTCGTGAAGACCCTGACTGGTAAGACCATCACTCTCGAAGTGGAGCCGAGTGACACCATTGAGAATGTCAAGGCAAAGATCCAAGACAAGGA	HK
UBE2C	NM_007019.2	NM_007019.2:445	GTCTGCCCTGTATGATGTCAGGACCATTCTGCTCTCCATCCAGAGCCTTCTAGGAGAACCCAACATTGATAGTCCCTTGAACACACATGCTGCCGAGCTC	
UBE2T	NM_014176.1	NM_014176.1:50	GTGTCAGCTCAGTGCATCCCAGGCAGCTCTTAGTGTGGAGCAGTGAACTGTGTGTGGTTCCTTCTACTTGGGGATCATGCAGAGAGCTTCACGTCTGAAG	

HK housekeeping gene

Supplementary table 2. Demographic data of breast cancer patients in the study.

MenCER (M) or Vietnamese (V) study	Age in years	Number of positive lymph nodes	Tumour grade	Tumour size	Tumour type	PgR status
V	44	0	2	20	IDC	+
V	47	1	1	20	IDC	-
V	49	0	2	30	IDC	+
V	33	2	2	30	IDC	+
V	36	1	2	12	IDC	+
V	36	1	2	15	IDC	+
V	39	0	2	15	IDC	+
V	41	0	1	41	IDC	+
M	40	0	3	50	IDC	+
M	40	0	3	38	IDC	+
M	43	0	1	25	IDC	+
M	37	0	3	11	IDC	+
M	34	0	2	10	IDC	+
M	49	2	1	17	IDC	+
M	49	1	2	41	IDC	+
M	43	0	2	10	IDC	+
M	45	0	2	32	IDC	+
M	42	0	3	35	IDC	+
M	48	0	3	30	IDC	+
M	46	0	3	53	IDC	+
M	49	1	2	50	IDC	+
M	44	0	2	58	lobular	+
M	49	0	2	15	IDC	-
M	49	0	1	9	IDC	+

M	44	0	1	15	IDC	+
M	44	0	3	10	lobular	+
M	43	0	2	32	IDC	-
M	43	0	2	25	IDC	+
M	41	1	3	34	IDC	+
M	47	0	2	24	IDC	+

IDC intraductal carcinoma, *PgR* progesterone receptor
